# Masseter Vestibular Evoked Myogenic Potentials (M-VEMPs) in Vestibular Neuritis

**DOI:** 10.3390/audiolres15030063

**Published:** 2025-05-26

**Authors:** Francesco Comacchio, Giulia Zattoni, Valerio Maria Di Pasquale Fiasca, Paola Magnavita, Barbara Bellemo, Elena Fasanaro, Elisabetta Poletto

**Affiliations:** 1 Otorhinolaryngology Unit, Regional Vertigo Specialized Centre, Sant’Antonio Hospital, University Hospital of Padua, 35121 Padua, Italy; 2 Department of Neurosciences, Section of Otolaryngology, University Hospital of Padua, 35121 Padua, Italy

**Keywords:** vestibular evoked myogenic potentials, masseter-VEMPs, M-VEMPs, MVEMPS, CVEMPS, OVEMPS, vestibular neuritis, acute unilateral vestibulopathy

## Abstract

**Introduction**: Masseter vestibular evoked myogenic potentials (M-VEMPs) are a recent tool for assessing a vestibulo-trigeminal pathway departing from the saccule, similarly to cervical VEMPs (C-VEMPs), that evaluate saccular function via the sternocleidomastoid muscle. M-VEMPs may offer a complementary diagnostic value in vestibular neuritis (VN). **Methods**: This retrospective study analysed M-VEMPs and C-VEMPs in 28 monolateral patients and 1 bilateral (30 ears) diagnosed with VN between 2023 and 2024. Diagnostic evaluation included video head impulse tests (VHIT), caloric tests, ocular VEMPs, and, in a few cases, electromyography (EMG) of the sternocleidomastoid muscle. M-VEMPs were elicited using 500 Hz tone bursts at 97 dB nHL. Results were compared based on the topography of vestibular involvement and muscle response concordance. **Results**: M-VEMPs were always present in patients with superior VN and intact saccular function, showing consistent results with normal C-VEMPs. In some cases, with saccular dysfunction, M-VEMPs were preserved despite the absence of C-VEMPs, suggesting greater robustness. One patient with herpes zoster (HZ) involving both the VIII and trigeminal nerves showed absent M-VEMPs, indicating trigeminal pathway involvement. Edentulous patients showed reduced or absent M-VEMPs due to compromised masseter muscle electromyography activity. **Conclusions**: M-VEMPs are reliable and often concordant with C-VEMPs in VN but may reveal additional diagnostic information in discordant or complex cases. They are particularly useful in identifying trigeminal involvement but are limited in patients with poor masseter muscle function. Further studies are needed to clarify their full diagnostic potential.

## 1. Introduction

Vestibular evoked myogenic potentials (VEMPs) are well-established electrophysiologic tools that explore the otolithic responses induced by sound, vibration or galvanic stimulation. Ocular VEMPs (O-VEMPs) investigate the utricular ocular pathways through the extraocular muscles, whereas cervical VEMPs (C-VEMPs) investigate the saculocollic reflex through the sternocleidomastoid muscle.

It has long been recognised that otolithic stimulation of other muscles, such as the gastrocnemius, soleus, triceps, trapezius, and masseter, can elicit myogenic responses. Recently, the masseter vestibular evoked myogenic potentials have received a new interest [1,2,3,4,5,6,7]. Since the studies of Hickenbottom et al. [8], it has been demonstrated that vestibular stimulation can modulate a myogenic masseteric response. Meier-Ewert and associates [9] first reported an acoustic jaw reflex in a number of cranial muscles, including the masseters. The submaximal interference pattern of masseter electromyography (EMG) showed a dual bilateral inhibitory period (approximate latency 14 ms, duration 11 ms) following unilateral stimulation with high-intensity clicks or tones. The authors demonstrated that the acoustic nerve was its afferent reflex arc and proposed that it might be a local protective reflex, ruling out a vestibular component. Additionally, they discovered a subsequent potential, which seemed to be a component of the startle reflex. Responses of active masseter muscles were obtained in 2005 from normal hearing subjects [10] and consist of a bilateral biphasic potential p11/n21 wave at suprathreshold stimulation (>90 dB HL) called the vestibular masseter response (VMR) and a second potential with longer latency n16/p21 wave at low stimulation intensities (<80 dB HL) named the acoustic masseter response (AMR) [11]. The vestibular (saccular) origin of the first potential and the cochlear origin of the n16/p21 complex were established in patients [11]. The VMR represents the bilateral, electromyographic inhibitory response of activation of the vestibular masseter pathways that originate from the saccular maculae and are carried out via the inferior vestibular nerve, the ipsilateral medial nucleus, and then through a decussatory pathway to the trigeminal motor nuclei and the masseter muscle. The clinical role of VMR or M-VEMPs was studied in multiple sclerosis [12], Parkinson’s disease [13], REM sleep disorders [14], diabetes mellitus [15], Meniere’s disease [16], migraine [17], vestibular migraine [18], and motion sickness [19], but there are very few data on the role of M-VEMPs in the analysis of the peripheral vestibular system and, specifically, in vestibular neuritis (VN). A recent paper by Rajesh and Neupane [20] described the results of M-VEMPs recordings in three patients affected by VN. The present study is the first retrospective analysis of the results of M-VEMPs recording and its comparison with C-VEMPs in a more consistent group of patients suffering from VN.

## 2. Materials and Methods

A group of patients with acute vestibular loss compatible with VN were enrolled in this retrospective investigation. The individuals were outpatients assessed between 2023 and 2024 at the Otolaryngology Unit of the Regional Vertigo Specialized Centre of Sant’ Anthony Hospital of University Hospital of Padua. All patients were assessed within 30 days since the beginning of their symptoms. Those patients evaluated after this period, were excluded from the analysis. Informed consent was obtained by each participant in this study. The study was authorised by the Padova Ethics Committee (protocol code 6192/AO/25 accepted on 23 January 2025) for human subjects research and carried out in compliance with the Declaration of Helsinki. According to the Committee for the Classification of Vestibular Disorders of the Bárány Society [21], acute unilateral vestibulopathy/VN is diagnosed as an acute vestibular syndrome based on a patient’s history, bedside and laboratory examination evidence of reduced VOR function on the side opposite the direction of the fast phase of spontaneous nystagmus, and an acute unilateral loss of peripheral vestibular function with spinning vertigo that lasts for at least 24 h without evidence of acute central or acute audiological symptoms or signs. An additional criterion for exclusion is the existence of conductive or mixed hearing loss.

Fitzgerald Hallpike’s bithermic caloric assessment using an ICS device and spontaneous and positional nystagmus recording were performed on all patients during infrared computerised videonystagmography (VNG). An Interacoustic Eyes See Cam device was used to conduct a video head impulse test (VHIT) for the six canals with Himps and Shimps protocols. When the amplitude was greater than 50°, pathological covert and overt saccades were evaluated, and pathological gain of VOR for single canals was considered with a minor of 0.8.

An Interacoustic Eclipse instrument was used to evaluate VEMPs. The participants sat up straight and comfortably in their chairs. Tone bursts at 500 Hz (rise/fall period of 4–2–4 ms) elicited air-conducted M-VEMPs. Using Radioear DD45 S earphones, stimuli were given monoaurally at 5.1 Hz with filters of 0.5–1500 Hz and an intensity of 97 dB nHL. C-VEMPs and O-VEMPs were elicited using air-conducted tone bursts at 500 Hz (rise/fall period of 4–2–4 ms). Using Radioear DD45 S earphones, stimuli were given monoaurally at 7.7 Hz, with filters of 0.5–1500 Hz for C-VEMPs and 0.5–1000 Hz for O-VEMPs, and an intensity of 97 dB nHL.

For M-VEMPs, the reference electrode was positioned over the zygomatic arch (2 cm above the active electrode), the active electrode was positioned on the bottom portion of the masseter muscle, and the forehead was ground. Interelectrode and absolute impedance were kept below 2 kOhms and 5 kOhms, respectively. Three control recordings were made to improve the repeatability assessment. The placement of the electrodes was consistent among all the participants.

Based on the VHIT data, the topographical location of the lesion was identified. Gain reduction or overt or covert saccades in the posterior semicircular canal (PSC) were regarded as indicators of involvement of the inferior vestibular nerve.

## 3. Results

The epidemiological information for our sample group is shown in Table 1. Twenty-nine patients (seventeen males and twelve females) were chosen for the analysis for thirty ears (one had a sequential VN and was evaluated twice). Age range: 27–90 years; mean age: 60.1 years. Twelve patients had a classical superior vestibular nerve neuritis, four had superior vestibular nerve neuritis plus saccular impairment, ten had supero-inferior vestibular nerve involvement, and three had supero-inferior VN with saccular sparing, according to the topographical site of the lesion. An isolated acute utricular dysfunction was diagnosed in only one case.

**Figure 1:**
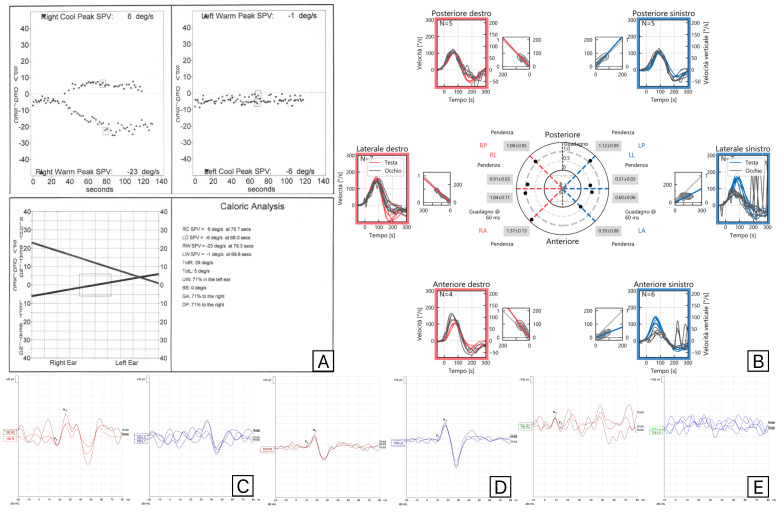
Vestibular assessments (Caloric Tests (**A**), Video Head Impulse test (**B**), C-VEMPs (**C**), M-VEMPs (**D**), and O-VEMPs (**E**)) results in a patient with involvement of the left superior vestibular nerve and saccule, showing normal M-VEMPs and absent C-VEMPs. Red is used for right-side evaluations and blue for left-side evaluations.

Canal paresis at caloric tests, decreased anterior and lateral semicircular canal gain at VHIT, impairment of O-VEMPs, and normal posterior semicircular canal VHIT gain and C-VEMPs were the findings of patients with classical superior vestibular nerve dysfunction. Both C-VEMPs and M-VEMPs were always elicited in this patient’s group. All cases presented latency and amplitude values within normal limits. In SVN neuritis cases, M-VEMPs and C-VEMPs were always concordant. In the four patients with SVN and saccular involvement, C-VEMPs were absent in all cases, but two presented normal M-VEMPs, as reported in Figure 1.

An EMG of the sternocleidomastoid muscle was performed on all individuals with normal M-VEMPs but no C-VEMPs, and the results were within normal limits. At three months follow-up, both patients completely recovered C-VEMPs. The M-VEMPs and C-VEMPs of the sole patients with acute utricular dysfunction were normal.

**Figure 2:**
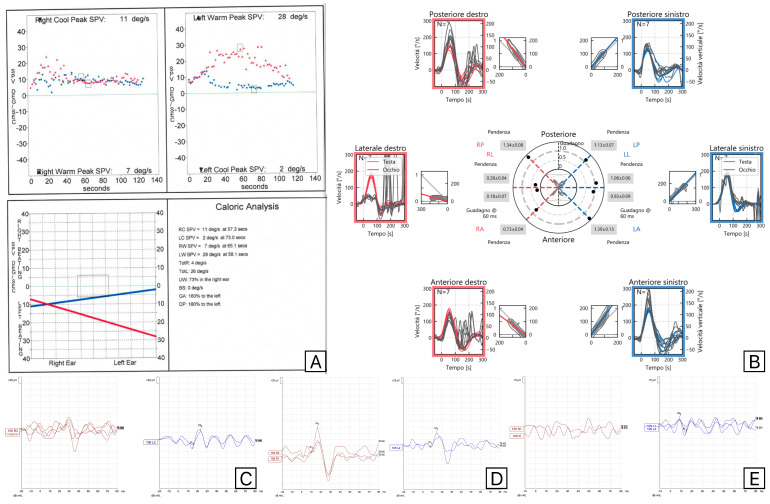
Results of vestibular tests (Caloric Tests (**A**), Video Head Impulse test (**B**), C-VEMPs (**C**), M-VEMPs (**D**), and O-VEMPs (**E**)) in a patient who showed a right supero-inferior VN, with normal M-VEMPs and abnormal responses at the Video Head Impulse Test in Posterior Semicircular Canal and absent C-VEMPs. Red is used for right-side evaluations and blue for left-side evaluations.

There was more discordance between C-VEMPs and M-VEMPs in the ten individuals with supero-inferior VN. M-VEMPs were unexpectedly present and within the usual range of A/L values in two patients with PSC involvement at VHIT and no C-VEMPs. The results of the vestibular tests performed on one of these patients are reported in Figure 2.

**Figure 3:**
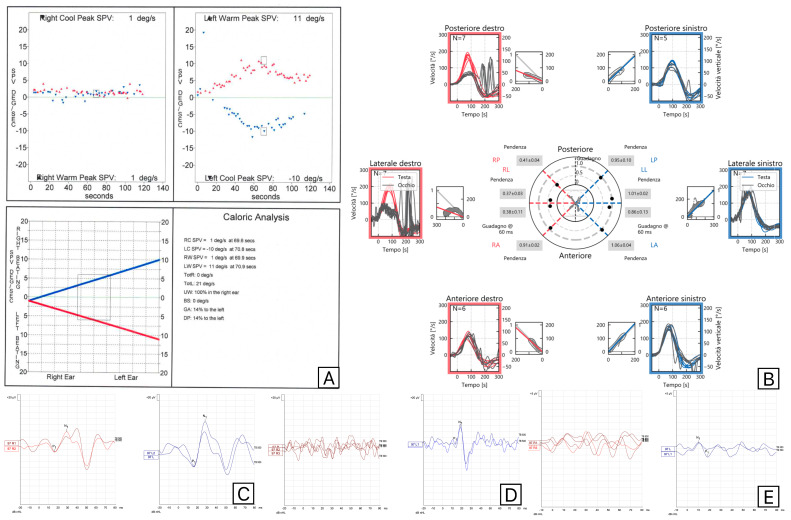
Results of vestibular tests (Caloric Tests (**A**), Video Head Impulse test (**B**), C-VEMPs (**C**), M-VEMPs (**D**), and O-VEMPs (**E**)) performed on a patient with a Herpes Zoster reactivation involving the right superior vestibular nerve and trigeminal nerves. Red is used for right-side evaluations and blue for left-side evaluations.

One patient presented a reduction in C-VEMPs and impairment of PSC VHIT gain but complete absence of M-VEMPs. This was an interesting case with a herpes zoster (HZ) infection of the superior vestibular nerve and trigeminal nerve. The patient presented with right otalgia and subsequent development of vesicles (on the pinna, retroauricolar, mastoid and temporal regions, cheek and intraorally in the homolateral soft palate), fever, VII cranial nerve paralysis (III grade on House–Brackmann scale) and vertigo. The results of its vestibular tests are reported in Figure 3.

The complete absence of M-VEMPs was attributed to the impairment of trigeminal pathway. Three patients exhibit supero-inferior involvement at VHIT. Still, their saccular function at C-VEMPs and M-VEMPs was completely normal. A different subgroup of patients had no M-VEMPs, and the amplitude of C-VEMPs on the affected side was reduced. We did not classify these individuals as discordant because they were all edentulous and had dental prostheses on the afflicted side.

## 4. Discussion

VN is characterised by acute spontaneous vertigo without hearing loss and central signs. It is considered the third most common peripheral vestibular disorder, after benign paroxysmal positional vertigo and Ménière disease. A separate lesion involving the contralateral nerve has been reported in 1–4% of patients [22,23,24,25], although this is usually unilateral. Additionally, a case of sequential vestibular supero-inferior neuritis is found in this series. Viral infection or vascular ischemia are considered the possible etiopathologic cause of VN. VN is in some cases preceded by an upper respiratory tract infection, and the cochleo-vestibular nerve involvement during HZ oticus is well-known. In our sample, a case presented with an HZ oticus with concurrent trigeminal impairment. The patient improved with therapy, with almost complete regression of acute symptoms, but developed a chronic imbalance, which was managed with vestibular rehabilitation, and post-herpetic trigeminal neuralgia. The concomitant involvement of both V and VIII cranial nerves is not frequent but has already been described in the literature [26,27].

The absence of M-VEMPs with partial impairment of C-VEMPS and PSC demonstrates the involvement of the vestibulo-trigeminal pathway. This is the first demonstration of this type of lesion using the M-VEMPs. Studies in humans also support the etiopathogenetic role of Herpes viruses but point to histopathological changes in both the vestibular nerve and labyrinth [28,29]. Herpes Simplex Virus (HSV) type 1 has also been detected in the vestibular ganglion in experimental studies in mice [30]. The authors observed that the HSV spread to Schwann and satellite cells only in the geniculate ganglion but not in the vestibular ganglion due to the protective effect of loose myelin in the latter. Vestibular ganglion neurons have a unique structure, with a loose myelin sheath instead of the satellite cell sheath that is seen in other ganglia. The lack of myelin seems to explain the relatively rare infection of vestibular nerves due to HSV as compared to facial nerve palsy. At the same time, ischemic lesions have also been demonstrated as possible causes of VN [31,32,33,34]. Recently, we were able to show a case of ossification of PSC following occlusion of the posterior vestibular artery, which mimicked an inferior VN, and a case of sudden bilateral acute neuropathy during myocardial infarction, which was caused by failure in hemodynamic perfusion of both anterior vestibular arteries [25]. Generally, three forms of vestibular nerve involvement are present in VN: superior vestibular nerve involvement, the most frequent, a complete impairment of the superior and inferior vestibular nerves, and a less frequent form involving only the inferior vestibular nerve. In our case series, the superior vestibular nerve presentation was the most prevalent presenting pattern, according to data from Navari et al. [35] and Yacovino et al. [36]. It is commonly recognised that the superior branch of the vestibular nerve is more susceptible. A number of theories were put forth to explain this pattern, including a direct viral invasion through the facio-vestibular anastomosis that specifically affected the superior division—the superior division’s bony channel is narrower and longer than the inferior division’s, making the former more susceptible to compression inside the channel from oedema [37]—and a double innervation of the PSC that provides greater resistance to injury of the inferior portion of the labyrinth [28]. A few subgroups have been identified with sparing or involving the saccule or utricular function as a result of advancements in otoneurological diagnostic tools. These subgroups do not always correspond with the classical topographical pattern of VN or the classical innervation of the labyrinth, which makes it more difficult to distinguish between intralabyrinthine and neural involvement of the lesion. Our results unequivocally show that in certain patients—those with normal PSC function and pathological C-VEMPs, or those with pathological PSC function and normal C-VEMPs—there is a dissociation pattern between PSC involvement and saccular function. Other authors have reported similar contradictory findings in their series [35,36,38,39]. The presence of a branch of the anterior vestibular artery supplying the superior part of the saccule and the Voit’s anastomosis, which allows a portion of the saccular afferent neurons to travel via the superior vestibular nerve, has been investigated as potential explanations.

Therefore, in cases of anterior vestibular artery obstruction or superior vestibular nerve neuritis, the saccule may be affected through these pathways. M-VEMPs were fully normal in two of the four patients with saccular impairment that C-VEMPs documented. In superior vestibular nerve involvement, in the event of saccular sufferance, M-VEMPs demonstrated greater robustness than C-VEMPs. In these situations, the full recovery of cervical potentials at follow-up and a normal sternocleidomastoid muscle EMG allows for the rule out of peripheral muscular contraction issues. There was congruence between M-VEMPs and C-VEMPs in cases with saccule sparing and tricanalar involvement. In the only patient with vestibular impairment studied by Deriu et al. [11], only the AMR was observed at 16 ms, while the VMR was absent. The three cases reported by Rajesh and Neuphane [20] affected by supero-inferior VN exhibited concordant results between C-VEMPs and M-VEMPs. In more central pathologies such as migraine and vestibular migraine, concordant abnormalities in latency and amplitude were observed in C and M-VEMPs [18]. M-VEMPs were more significantly impaired than C-VEMPs in studies conducted on patients affected by Parkinson’s disease [13], where a statistically significant decrease in amplitude of M-VEMPs was observed, while C-VEMPs were normal. These findings may reflect the upper brainstem involvement of M-VEMPs more than C-VEMPs.

Uffer and Hegemann [40] considered the VN as an intralabyrinthine lesion when the pattern of involvement is not following the innervation pattern and found that the intralabyrinthine pattern is more frequent than the classical neuritic pattern. Murofushi et al. [41] used the galvanic VEMPs stimulation in a group of “VN” patients and demonstrated that some subjects had labyrinthine involvement. In contrast, others had a neural pattern or a coexistence of the two, and proposed the term of neurolabyrinthitis as more appropriate. According to the standards of Uffer and Hegemann [40], most instances (20 out of 30) in our collection exhibit a conventional neuritic pattern, while 10 exhibit an intralabyrinthine pattern. In certain cases, with saccular involvement, the discrepancy between M-VEMPs and C-VEMPs appears to raise additional questions about the lesion site or the M-VEMPs origin.

Sound is a natural stimulus for both cochlear and saccular receptors. Deriu et al. [10] showed that the masseter muscle responses to loud click stimulation are made up of two partially overlapping short-latency reflexes, a p11/n15 wave and a p16/n21 wave, in both deaf participants and patients with vestibular impairment and normal hearing. Their distinct physiological characteristics imply that they are caused by the activation of distinct inner ear receptors: cochlear receptors for the p16/n21 response and saccular receptors for the p11/n15 response. Considering their similar stimulation patterns and most likely common origin in the saccular macula (though some recent research has suggested that the C-VEMPs have a non-exclusively saccular origin [42]), C-VEMPs seem to be more vulnerable to minor saccular damage than M-VEMPs, which have demonstrated more stability in our series. This preliminary observation requires further confirmation. Do C-VEMPs have a greater role in the fibres that pass through the anastomosis of Voit’s? Are there distinct patterns of saccular macula stimulation? To explain these initial findings, additional neurophysiological research and larger case series are required.

The lack of decrease in response in certain cases of dentary implantation or edentulia is a limitation of M-VEMPs that we observed in clinical practice and in this case series. This observation is now the subject of a specific study at our centre. When assessed using ultrasonography [43], it has been demonstrated that edentulous patients have significantly thinner masseter muscles than dentate subjects. This difference may account for the difficulties in evoking M-VEMPs in certain edentulous patients, probably for the documented reduction in M-VEMPs amplitude with the reduction in EMG activity [44].

Future developments in research on M-VEMPs and their use will certainly include more in-depth studies of their relationship with C-VEMPs, comparing the accuracy of the two techniques in detecting saccule dysfunctions and their efficacy over time. The main limitation of this method, the alterations in the dentition, is also an area of study that will have to be explored in depth. Furthermore, our study is the first retrospective analysis of this technique in the analysis of VN. The quantitative analysis of the diagnostic accuracy of this technique is not currently available in the literature and needs to be tested. Likewise, a quantitative comparison with diagnostic features of C-VEMPs has to be performed: this will be the object of a subsequent study.

## 5. Conclusions

M-VEMPs are a reliable diagnostic tool to explore the vestibulo-trigeminal pathway. They always show a concordant pattern with C-VEMPs in VN when the latter are normal but present some degree of discordance when C-VEMPs are impaired, showing a more robust behaviour in case of lesion. Further studies are required to confirm their clinical role and to determine whether the presence of M-VEMPS can help in identifying minor damage and the recovery of saccular function. In the only case with documented trigeminal HZ involvement, M-VEMPs were of great utility in the monitoring of such a lesion, showing the involvement caused by the virus on both the vestibular and trigeminal pathways.

## Figures and Tables

**Table 1:** Epidemiological characteristics of the included patients.

ID	Age	Sex	Impairment	Side	Herpes Zoster
1	80	F	SIVN	R	
2	80	F	SIVN	L	
3	60	F	SIVN	R	+
4	75	M	SVN with ipsilateral saccule deficit	L	
5	69	F	SIVN	R	
6	73	F	SIVN	R	
7	59	M	SIVN	L	
8	40	F	SVN with ipsilateral saccule deficit	L	
9	38	F	SVN	L	
10	45	M	SVN	L	
11	79	M	SIVN	R	
12	63	M	SVN	R	
13	76	M	SVN	L	
14	42	F	SVN	R	
15	43	M	SVN	R	
16	64	M	SVN	R	+
17	90	F	SIVN	L	
18	51	M	SIVN with ipsilateral saccule sparing	R	
19	64	M	SIVN with ipsilateral saccule sparing	L	
20	27	M	SVN	R	
21	74	F	Utricle	L	
22	65	F	SVN	L	
23	62	M	SVN with ipsilateral saccule deficit	R	
24	57	M	SVN with ipsilateral saccule deficit	L	
25	47	M	SIVN with ipsilateral saccule sparing	L	
26	31	F	SVN	L	
27	55	M	SVN	R	
28	77	M	SVN	R	
29	54	F	SIVN	R	
30	63	M	SIVN	L	+

F: female; M: male; SVN: superior VN; SIVN: supero-inferior VN; L: left; R: right. Patients affected by Herpes zoster were indicated with “+”.

## Data Availability

Data are unavailable due to privacy or ethical restrictions.
